# Efficacy and safety of intraperitoneal ropivacaine in pain management following laparoscopic digestive surgery: A systematic review and meta-analysis of RCTs

**DOI:** 10.1097/MD.0000000000038856

**Published:** 2024-07-19

**Authors:** Mohamed Aziz Daghmouri, Mohamed Ali Chaouch, Benjamin Deniau, Laurent Benayoun, Bassem Krimi, Amine Gouader, Hani Oweira

**Affiliations:** a Department of Anesthesia, Montreuil Intercommunal Hospital Center, Montreuil, France; b Department of Visceral and Digestive Surgery, Fattouma Bourguiba Hospital, University of Monastir, Monastir, Tunisia; c Department of Anesthesiology, Critical Care and Burn Unit, University Hospital Saint-Louis-Lariboisière, AP-HP, Paris, France; INSERM UMR-S 942, Cardiovascular Markers in Stress Condition (MASCOT), Université de Paris Cité, Paris, France; d Department of Surgery, Perpignan Hospital Center, Perpignan, France; e Department of Anesthesiology, Perpignan Hospital Center, Perpignan, France; f Department of Surgery, Universitäts Medizin Mannheim, Heidelberg University, Mannheim, Germany.

**Keywords:** analgesia, intraperitoneal instillation, laparoscopic surgery, meta-analysis, ropivacaine

## Abstract

**Background:**

Managing postoperative pain effectively with an opioid-free regimen following laparoscopic surgery (LS) remains a significant challenge. Intraperitoneal instillation of ropivacaine has been explored for its potential to reduce acute postoperative pain, but its efficacy and safety are still under debate. This study aimed to evaluate the efficacy and safety of intraperitoneal instillation of ropivacaine for acute pain management following laparoscopic digestive surgery.

**Methods:**

We used PRISMA 2020 and a measurement tool to assess systematic reviews 2 guidelines to conduct this review. The random-effects model was adopted using Review Manager Version 5.4 for pooled estimates.

**Results:**

We retained 24 eligible RCTs involving 1705 patients (862 patients in the intraperitoneal instillation group and 843 patients in the control group). The intraperitoneal instillation group reduced total opioid consumption during the first 24 hours postoperatively (MD = −21.93 95% CI [−27.64, −16.23], *P* < .01), decreased pain scores at different time (4 hours, 8 hours, 12 hours and 24 hours), shorter the hospital stay (MD = −0.20 95% CI [−0.36, −0.05], *P* < .01), reduced the postoperative shoulder pain (MD = 0.18 95% CI [0.07, 0.44], *P* < .01), and decreased postoperative nausea and vomiting (MD = 0.47 95% CI [0.29, 0.77], *P* < .01).

**Conclusion:**

Intraperitoneal instillation of ropivacaine appears to be an effective component of multimodal pain management strategies following laparoscopic digestive surgery, significantly reducing opioid consumption and improving postoperative recovery markers. Despite these promising results, additional high-quality trials are needed to confirm the efficacy and safety of this approach.

**Registration:**

The registration number at PROSPERO was CRD42021279238.

## 1. Introduction

Managing pain effectively after laparoscopic surgery (LS) using an opioid-free approach presents a significant challenge. Laparoscopy is deemed to be a minimally painful and efficient method, even for complex procedures, enhancing postoperative recovery.^[1,2]^ Nevertheless, reports of postoperative discomfort persist. Ekstein et al^[3]^ found that LS was associated with severe pain and a higher requirement for analgesics within the first 4 hours post-surgery compared to open surgery. This phenomenon is primarily attributed to peritoneal irritation, which causes postoperative shoulder pain.^[3,4]^ Consequently, various strategies, including anti-inflammatory medications, reduced pneumoperitoneum pressure, transversus abdominis plane block, and wound infiltration, have been suggested to alleviate this discomfort.^[5,6]^ Yet, the optimal approach remains a subject of debate. Therefore, opioids are still utilized despite their numerous side effects, such as postoperative nausea and vomiting (PONV), excessive sedation, and extended hospital stays (length of stay, LOS).^[7,8]^ In the context of the enhanced recovery after surgery multimodal strategy, the intraperitoneal instillation of ropivacaine has been explored in several studies, yielding mixed outcomes, hence its effectiveness is still contested.^[6]^ Other agents like bupivacaine or tramadol have also been employed,^[9,10]^ but ropivacaine is preferred for its lower cardiotoxicity, allowing for higher dosages.^[11]^ This systematic review and meta-analysis of randomized controlled trials (RCTs) seeks to evaluate the efficacy and safety of intraperitoneal ropivacaine instillation for acute pain management following laparoscopic digestive surgery.

## 2. Methods

We conducted this systematic review and meta-analysis according to the Preferred Reporting Items for Systematic Review and Meta-analysis (PRISMA) guidelines 2020 and A MeaSurement Tool to Assess systematic Reviews 2 (AMSTAR 2; assessing the methodological quality of systematic reviews) Guidelines. Therefore, we did not submit a review protocol previously before the completion of the evaluation. This study was registered in PROSPERO under the CRD42021279238.

### 2.1. Electronics searches

We performed the electronic investigation of the relevant literature on August 15, 2021, for the publications during the last 2 decades. We did not use language restrictions. We sought trials in the Cochrane Library’s Controlled Trials Registry and systematic review database, Embase, National Institutes of Health PubMed/MEDLINE, and Google Scholar databases. We used the following Keywords: “analgesia,” “pain management,” “intraperitoneal,” “ropivacaine,” “local anesthetic,” “LS,” “laparoscopy,” “visceral surgery,” “cholecystectomy,” “appendectomy,” “colectomy,” “sleeve gastrectomy,” “randomized-controlled trials,” and “placebo.” We checked the reference list of relevant reviews for eligible clinical trials.

### 2.2. Inclusion criteria

We retained only RCTs comparing intraperitoneal ropivacaine infusion with placebo or no intraperitoneal infusion for postoperative pain management following laparoscopic digestive surgery for adults (>18 years old).

Only articles published in peer-reviewed journals were considered. Data from controlled clinical trials, noncomparative studies, review articles, editorial letters, abstract only, comments, and case series (<10 cases) were excluded.

### 2.3. Studies populations

Patients having laparoscopic digestive surgery included in RCTs.

### 2.4. Intervention group

Intraoperative intraperitoneal ropivacaine instillation or injection.

### 2.5. Control group

Another regional analgesic technique different from placebo.

### 2.6. Outcomes measures

The primary outcome was total opioid consumption in IV morphine equivalent during the first 24 hours postoperatively.

The secondary outcomes were visual analogue pain scores (VAS) at rest and effort at different periods (4 hours H4, 8 hours H8, 12 hours H12 and 24 hours H24), PONV, length of hospital stay, frequency of right shoulder pain and postoperative adverse events (convulsions, wound infections, and intra-abdominal collections).

### 2.7. Study selection

Two authors independently reviewed all abstracts. We retained all studies accompanied by the full text that met the inclusion criteria. Disagreements were resolved by discussion after consulting a 3rd member of the review team.

### 2.8. Data extraction

Two authors extracted the data independently (MAD and MAC), and the senior authors (HO) settled the disparities after discussion. Studies included were fully matched for the 1st author’s name, year of publication, country, body mass index, sample size (intraperitoneal ropivacaine group and control group), age, sex ratio, administration protocol of ropivacaine, type of LS, supplemental analgesic, follow-up and CONSORT scale.

### 2.9. Missing data

We contacted authors by e-mail in the occurrence of unclear bias domains or missing primary outcomes information of our meta-analysis. If the data were not reported numerically, we extracted it from figures.

### 2.10. Assessment of studies quality and risk of bias assessment

All studies that met the selection criteria were appraised independently by 2 authors (MAD and MAC). We used the CONSORT (Consolidated Standards of Reporting Trials) scale for RCT quality assessment.^[3]^ We excluded studies with a score < 14/25. We used the Cochrane tool for bias assessment to assess the risk of bias in randomized trials (RoB2).^[4]^ We evaluated the bias in 6 distinct domains (randomization process, deviations from intended interventions, the bias in the measurement of outcome, bias to missing outcome data, bias in selecting the reported results, and overall bias). Within each domain, 1 or more signaling questions lead to judgments of “low risk of bias,” “some concerns,” or “high risk of bias.” The results were presented in the forest plot of each outcome.

### 2.11. Handling continuous data

Continuous data were analyzed using Review Manager 5.3.5 statistical package from Cochrane collaboration for meta-analysis.^[5]^ When mean and standard deviation were not reported, they were estimated from the provided interquartile range (IR) and median based on the formula described by Hozo et al^[6]^ If the sample size was >25 patients, then the mean was equal to the median. In addition, standard deviation was calculated as IR/4 for a sample size < 70 patients and IR/7 for a sample size > 70 patients.

### 2.12. Assessment of heterogeneity

To assess heterogeneity, 3 strategies were used:

1. The Cochrane Chi² test (*Q* test), Tau², which is the variance of true effects, and 95% predictive interval (index of dispersion) were used to estimate the degree of heterogeneity.^[7]^ We calculated the predictive intervals using a comprehensive meta-analysis. Values <25% indicated no heterogeneity, values between 25% and 50% indicated moderate heterogeneity, and values >50% indicated substantial heterogeneity.
2. Graphical exploration with funnel plots.^[8]^
3. Sensitivity analysis with a subgroup analysis when applicable.^[9]^

#### 2.12.1. Summary of findings

Two authors (MAD and MAC) independently assessed the certainty of the evidence. We used the Grading of Recommendations Assessment, Development and Evaluation.^[10]^ We considered the study limitations constancy of effect, imprecision, indirectness, and publication bias. We assessed the certainty of evidence as high, moderate, low, or very low. If appropriate, we considered the following criteria for upgrading the evidence: large effect, dose-response gradient, and plausible confounding effect. We used the methods and recommendations described in Sections 8.5 and 8.7 and Chapters 11 and 12 of the Cochrane Handbook for Systematic Reviews of Interventions. We used GRADEpro GDT software to prepare the Summary of findings tables. We explain the reasons for downgrading or upgrading the certainty of included studies using footnotes with comments.

#### 2.12.2. Evaluation of effect size

We used the RevMan 5.3.5 statistical package from the Cochrane collaboration for meta-analysis.^[5]^ We selected the mean difference (MD) as an effective measure for continuous data. Odds ratios with 95% confidence intervals (95% CI) were calculated for dichotomous variables. The random-effects model was used, and the threshold of significance was fixed at 0.05.

## 3. Results

### 3.1. Literature search

**Figure 1.**
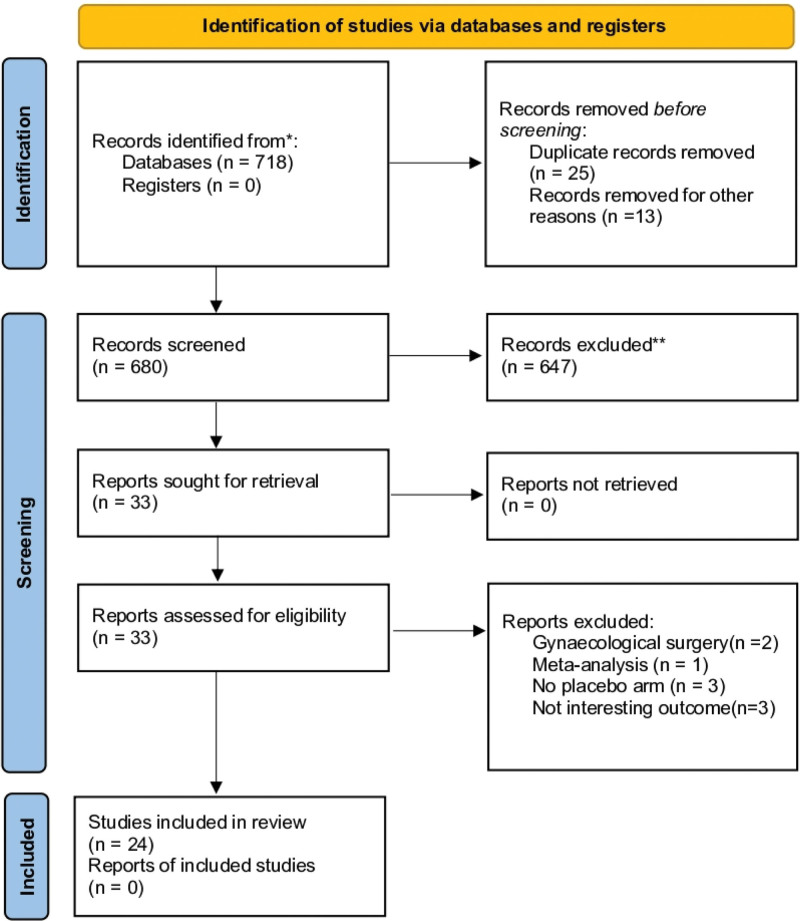
Flow diagram of included studies.

As initial research, we identified 680 papers from the electronic database, and after full-text checking, only 24 RCTs published between 2002 and 2021 were retained (Fig. 1). We excluded 9 studies for reasons: 2 studies assessed ropivacaine instillation following gynecological surgery,^[11,12]^ 3 trials did not report the outcome of interest,^[13–15]^ 1 study was a meta-analysis,^[16]^ and 3 studies compared intraperitoneal instillation to another analgesic technique.^[17–19]^ All the eligible RCTs were published as full papers in English. They involved 1705 patients (862 patients in the intraperitoneal instillation group and 843 in the control installation group). We reported studies ‘study characteristics with quality assessment and the risk of bias assessment in Tables 1 and 2, respectively (Supplementary Digital Content 1, http://links.lww.com/MD/N148).

**Table 1:** Studies characteristics.

Authors	Year	Country	Age/gender (M/F)	Sample size (Rop/CG)	Type of surgery	Duration of surgery	Ropivacaine protocol	Supplemental analgesic	Follow-up	CONSORT
Abet et al^[20]^	2016	France	47.8 (31/69)	100 (50/50)	Cholecystectomy	Rop: 56.1 ± 11.7 CG: 57.2 ± 16	Ropivacaine (7.5 mg/mL):10 mL at the vesicular level, 10 mL under the diaphragmatic cupola, infiltration of 10 mL in each 10 mm trocar opening and 5 mL in each 5 mm trocar	Paracetamol, ketoprofen, morphine	30 d	17/25
Cha et al^[21]^	2011	Korea	49.9 (17/23)	40 (20/20)	Cholecystectomy	Rop: 52.5 ± 26.8 CG: 50.0 ± 36.8	100 mL of ropivacaine solution (2 mg/kg) was infused intraperitoneally after the creation of the pneumoperitoneum	PCA morphine	48 h	16/25
Das et al^[22]^	2017	India	39.3 (35/25)	60 (30/30)	Cholecystectomy	Rop: 97.2 ± 32.4 CG: 106.8 ± 13.2	20 mL of ropivacaine 0.375% in the subdiaphragmatic supra-hepatic surface of the liver and 5 mL in the gallbladder fossa. Further, 10 mL was used for port-site infiltration	Paracetamol, diclofenac, tramadol	24 h	18/25
Gupta et al^[23]^	2002	Sweden	53.5	40 (20/20)	Cholecystectomy	Rop: 70 ± 18 CG: 61 ± 17	Ropivacaine 0.5% (total, 10 mL) was injected into the site of the incision and in all portals at the end of the surgery in all patients	Paracetamol	24 h	15/25
Ingelmo et al^[24]^	2013	Italy	57 (24/33)	57 (28/29)	Cholecystectomy	Rop: 46 ± 32 CG: 55 ± 38	intraperitoneal nebulization of ropivacaine 1% (3 mL; 30 mg) at the end of surgery just before the deflation of pneumoperitoneum	Paracetamol, PCA morphine	48 h	19/25
Kaushal-Deep et al^[14]^	2017	India	39	157 (77/80)	Cholecystectomy	Rop: 38 ± 12 CG: 38 ± 12	At the end of the surgery, intraperitoneal instillation of the solution was done using the irrigation apparatus in the GB fossa and under the right diaphragm	Paracetamol, tramadol	24 h	17/25
Kucuk et al^[25]^	2007	Turkey	49.5 (7/33)	40 (20/20)	Cholecystectomy	Rop:87 ± 9 CG: 84 ± 10	At the end of the surgical procedure:7 mL under each subdiaphragmatic area and 7 mL to the gallbladder bed of ropivacaine 150 mg	PCA morphine	24 h	14/25
Labaille et al^[26]^	2002	France	49 (6/20)	26 (14/12)	Cholecystectomy	Rop: 125 ± 17 CG: 130 ± 26	Two intraperitoneal injections: the 1st immediately after pneumoperitoneum and the 2nd at the end of the surgery of ropivacaine 0.25% (20 mL)	Paracetamol, morphine	24 h	15/25
Liu et al^[27]^	2015	China	43.8 (41/34)	75 (37/38)	Cholecystectomy	Rop: 24.5 ± 10.6 CG: 21.2 ± 7.6	After gallbladder extraction, ropivacaine was intraperitoneally injected into the surgical bed using a feeding tube through the right subcostal port	Paracetamol, PCA morphine	48 h	17/25
Mcdermott et al^[28]^	2015	Ireland	44.4 (36/41)	87 (40/47)	Cholecystectomy	Rop: 35 ± 17.5 CG: 35 ± 12.5	Aerosolized intraperitoneal ropivacaine: 5 mL of ropivacaine 1% before surgery and 5 mL before insufflation	Paracetamol, morphine	24 h	18/25
Gogos et al^[29]^	2007	Greece	56.7	40 (20/20)	Cholecystectomy	Rop: 45.5 ± 18.3 CG: 39.05 ± 12.9	40 mL of ropivacaine solution (2 mg/mL) was infused at the beginning of the procedure under the right hemidiaphragm	Ketoprofen, codeine	72 h	17/25
Yeh et al^[30]^	2014	Taiwan	52.5 (50/60)	110 (55/55)	Cholecystectomy	Rop: 84.2 ± 22.6 CG: 84.9 ± 28.7	200 mL of ropivacaine 1% before surgery. The 1st infusion was administered in the right subdiaphragmatic region and the 2nd infusion was administered in the left subdiaphragmatic region.	Morphine	24 h	19/25
Singh et al^[31]^	2013	India	39.2 (40/60)	100 (50/50)	Cholecystectomy	-	Before the removal of trocar at the end of the surgery: the surgeon sprayed 10 mL of solution into the hepato-diaphragmatic space, 5 mL in the area of the gallbladder, and 5 mL into the space between liver and kidney	Diclofenac	24 h	15/25
Kim et al^[32]^	2010	Korea	50.1 (16/24)	40 (20/20)	Cholecystectomy	Rop: 53.55 ± 9.41 CG: 51.35 ± 11.32	Intraperitoneal instillation of 2 mg/kg of ropivacaine diluted in 100 mL saline at the initiation of pneumoperitoneum	PCA morphine	48 h	15/25
Alevizos et al^[33]^	2020	Cyprus	36.3 (23/37)	60 (40/20)	Sleeve gastrectomy	Rop: 49.3 ± 13.6 CG: 51.4 ± 12.9	Incisional infiltration with 20-mL ropivacaine 0.5% and Intraperitoneal instillation was administered through a catheter after specimen removal in the subdiaphragmatic space over the gastro-esophageal junction and along the staple-line	PCA morphine	48 h	17/25
Tovar et al^[34]^	2016	Spain	45.5 (35/75)	110 (55/55)	Sleeve gastrectomy	Rop: 94.8 ± 22.34 CG: 92.9 ± 23.2	300 mg total of ropivacaine in 200 mL normal saline was instilled into the abdomen after surgical dissection, just before abdominal wall closure. Under direct visualization, the solution was delivered over the esophageal hiatus, over both anastomoses and in both subdiaphragmatic spaces	Metamizole, acetaminophen	24 h	20/25
Custovic et al^[35]^	2018	Bosnia	32.4	60 (30/30)	Appendectomy	-	5 and 10 mL 0.5% ropivacaine were injected under direct vision in the right iliac fossa area and around the stump of the appendix and trocar sites at the end of the procedure	PCA morphine	48 h	15/25
Huang et al^[36]^	2019	Australia	30.5 (39/47)	86 (43/43)	Appendectomy	-	0.1 mL/kg 1% ropivacaine with normal Saline a total of 20 mL in volume was injected into the 3-port sites: 50% in preperitoneal space before port insertion with the remaining 50% injected before skin closure	PCA morphine	24 h	18/25
Kang et al^[37]^	2010	Korea	38.1 (30/33)	63 (30/33)	Appendectomy	Rop: 54.73 ± 10.80 CG: 53.73 ± 13.75	Instillation of 2 mg/kg ropivacaine at the initiation of the pneumoperitoneum	PCA morphine	48 h	17/25
Thanapal et al^[38]^	2012	Malaysia	38.4 (29/43)	72 (40/32)	Appendectomy	–	The prepared solutions were administered into the peritoneum (right paracolic gutter, cecum and appendix site) immediately upon introduction of the 3rd port (trocar)	Paracetamol, PCA morphine	24 h	18/25
Duffield et al^[39]^	2018	Australia	65.7 (46/40)	86 (44/42)	Colectomy	Rop: 153 ± 12.9 CG: 162 ± 15	Intraoperative intraperitoneal ropivacaine 100-mg bolus both pre- and post-dissection and 20 mg/h continuous postoperative infusion for 48 h	Paracetamol + Parecoxib + Tramadol	45 d	18/25
Kahokehr et al^[40]^	2011	New Zealand	69.2 (23/37)	60 (30/30)	Colectomy	Rop: 105 ± 50 CG: 123 ± 49.8	Before any dissection, a 50 mL loading dose of 75 mg of ropivacaine was instilled to coat the peritoneum, abdominal viscera, and paracolic gutters. After the procedure was completed, 2 plastic infusion catheters with multiple pores were placed in the corresponding paracolic gutter for continuous infusion of ropivacaine	Paracetamol, NSAID, Tramadol	30 d	18/25
Park et al^[41]^	2010	Korea	58.25	40 (20/20)	Colectomy	Rop: 252.8 ± 72.50 CG: 282.5 ± 54.30	Immediately after creation of a pneumoperitoneum and placement of the first 2 trocars, 50 mL of ropivacaine solution was sprayed onto the bowel surface and mesentery	PCA morphine	48 h	17/25
Stephensen et al^[42]^	2018	Australia	68.0 (57/39)	96 (49/47)	Colectomy	–	At completion of the surgical resection but before removal of laparoscopic ports, patients received a bolus of ropivacaine 80 or 40 mg	PCA morphine	48 h	19/25

CONSORT = Consolidated Standards of Reporting Trials, CG = control group, F = female, M = male, NSAID = Nonsteroidal Anti-Inflammatory Drugs, PCA = patient controlled analgesia, Rop = ropivacaine.

**Table 2:** Risk of bias 2 assessment of the included studies.

Authors	Randomization process	Deviations from intended interventions	Bias in measurement of outcome	Bias to missing outcome data	Bias in selecting the reported results	Overall bias
Abet et al^[20]^	High risk	Some concerns	Low risk	Low risk	Low risk	High risk
Alevizos et al^[33]^	High risk	Some concerns	Low risk	Low risk	Some concerns	High risk
Cha et al^[21]^	Low risk	Low risk	Low risk	Low risk	Low risk	Low risk
Custovic et al^[35]^	Some concerns	High risk	Low risk	Low risk	Low risk	Some concerns
Das et al^[22]^	Low risk	Low risk	Low risk	Low risk	Low risk	Low risk
Duffield et al^[39]^	Low risk	Low risk	Low risk	Low risk	Low risk	Low risk
Gogos et al^[29]^	High risk	High risk	Low risk	Low risk	Low risk	High risk
Gupta et al^[23]^	High risk	High risk	Low risk	Low risk	Low risk	High risk
Huang et al^[36]^	Low risk	Low risk	Low risk	Low risk	Low risk	Low risk
Ingelmo et al^[24]^	Low risk	Low risk	Low risk	Low risk	Low risk	Low risk
Kahokehr et al^[40]^	Low risk	Low risk	Low risk	Low risk	Low risk	Low risk
Kang et al^[37]^	Low risk	Low risk	Low risk	Low risk	Low risk	Low risk
Kaushal-Deep et al^[14]^	Low risk	High risk	Low risk	Low risk	Low risk	High risk
Kim et al^[32]^	High risk	Some concerns	Low risk	Low risk	Low risk	High risk
Kucuk et al^[25]^	High risk	High risk	Low risk	Low risk	Low risk	High risk
Labaille et al^[26]^	High risk	Some concerns	Low risk	Low risk	Low risk	High risk
Liu et al^[27]^	Low risk	Low risk	Low risk	Low risk	Low risk	Low risk
Mcdermott et al^[28]^	Low risk	Low risk	Low risk	Low risk	Some concerns	Some concerns
Park et al^[41]^	High risk	High risk	Low risk	Low risk	Low risk	High risk
Singh et al^[31]^	Some concerns	Low risk	Low risk	Low risk	Low risk	Some concerns
Stephensen et al^[42]^	Low risk	Low risk	Low risk	Low risk	Low risk	Low risk
Thanapal et al^[38]^	Low risk	Low risk	Low risk	Some concerns	Some concerns	Some concerns
Tovar et al^[34]^	Some concerns	High risk	Low risk	Low risk	Low risk	High risk
Yeh et al^[30]^	High risk	Some concerns	Low risk	Low risk	Low risk	High risk

## 4. Primary outcome: total opioid consumption postoperatively

**Figure 2.**
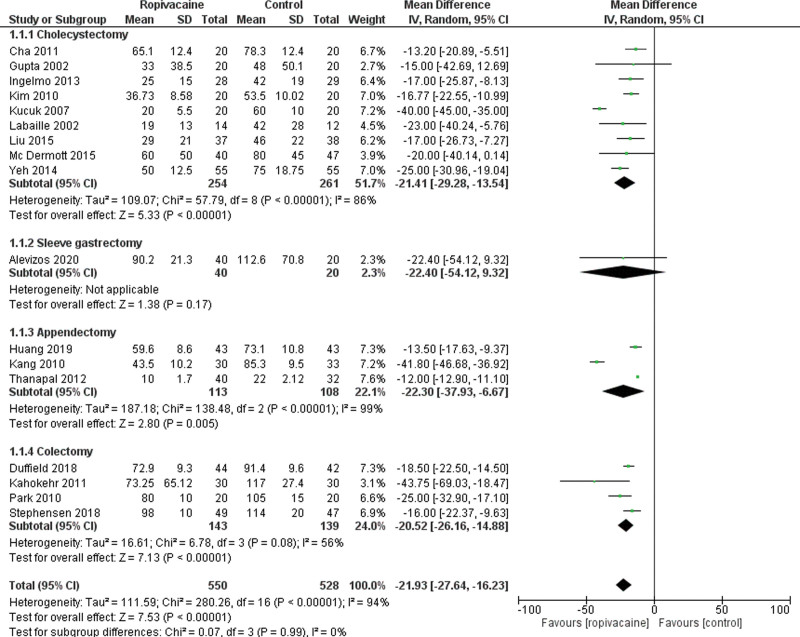
Forest plot for total opioid consumption. CI = confidence interval, SD = standard deviation.

A total of 17 studies reported data on total opioid consumption during the first 24 hours following surgery. Pooled results showed that the consumption was significantly lower in the intraperitoneal group (Moderate certainty of evidence; MD = −21.93 95% CI [−27.64, −16.23], *P* < .01). However, there was a high heterogeneity rate among the trials Tau^2^ = 111.59 (*I*² = 94%; Fig. 2). We performed a subgroup analysis comparing the different surgeries (cholecystectomy, sleeve gastrectomy, appendectomy, and colectomy). Nine studies reported opioid consumption in the cholecystectomy group.^[21, 23–28, 30, 37]^ There was a significantly lower consumption in the intraperitoneal group (MD = −21.41 95% CI [−29.28, −13.54], *P* < .01). Only one study reported opioid consumption following sleeve gastrectomy^[33]^ and showed similar results between both groups (MD = −22.40 95% CI [−54.12, 9.32], *P* = .17). Besides, 3 trials assessed data on opioid consumption in the appendectomy group.^[36–38]^ They included 113 patients in the intraperitoneal group versus 108 in the control group. There was a significantly lower consumption in the intraperitoneal group (MD = −22.30 95% CI [−37.93, −6.67], *P* < .01). Finally, following colectomy, the 4 included studies^[39–42]^ showed a significantly lower consumption of opioids in the intraperitoneal group (MD = −20.52 95% CI [−26.16, −14.88], *P* < .01).

### 4.1. Secondary outcomes

#### 4.1.1. Visual analogue pain scores

**Figure 3.**
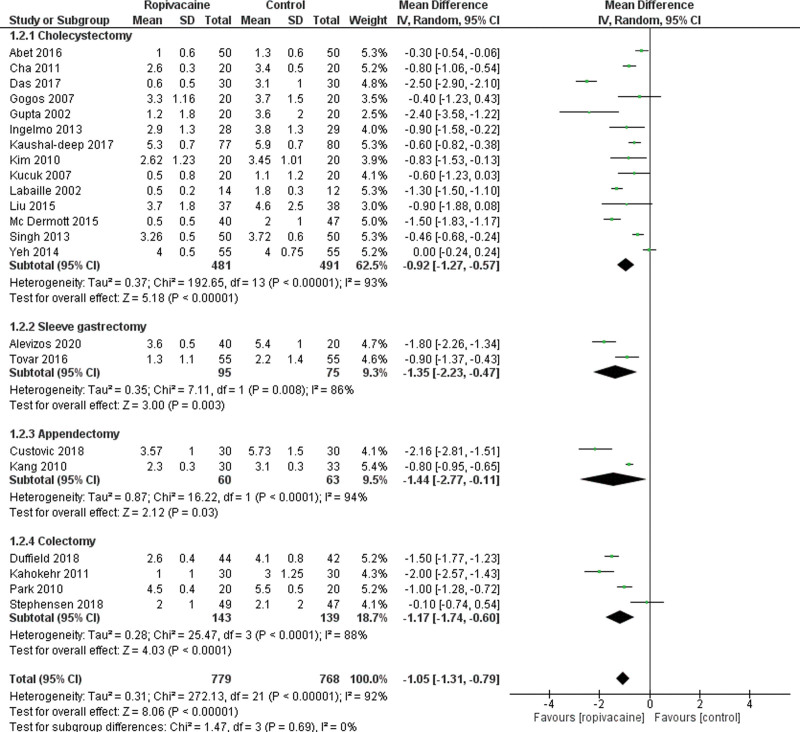
Forest plot for visual analogue scale at 4 hours. CI = confidence interval, SD = standard deviation.

Twenty-two studies assessed data on VAS score at H4 following LS.^[14, 20–24, 26–31, 33–35, 39–42]^ They included 779 patients in the intraperitoneal group versus 768 in the control group. Pooled results showed that VAS-H4 was significantly lower in the intraperitoneal group (Moderate certainty of evidence; MD = −1.05 95% CI [−1.31, −0.79], *P* < .01). There was moderate heterogeneity among the studies Tau^2^ = 0.31 (I^2^ = 92%). Subgroup analysis showed that VAS-H4 was still significantly lower in the intraperitoneal group despite the type of surgery (Fig. 3).

**Figure 4.**
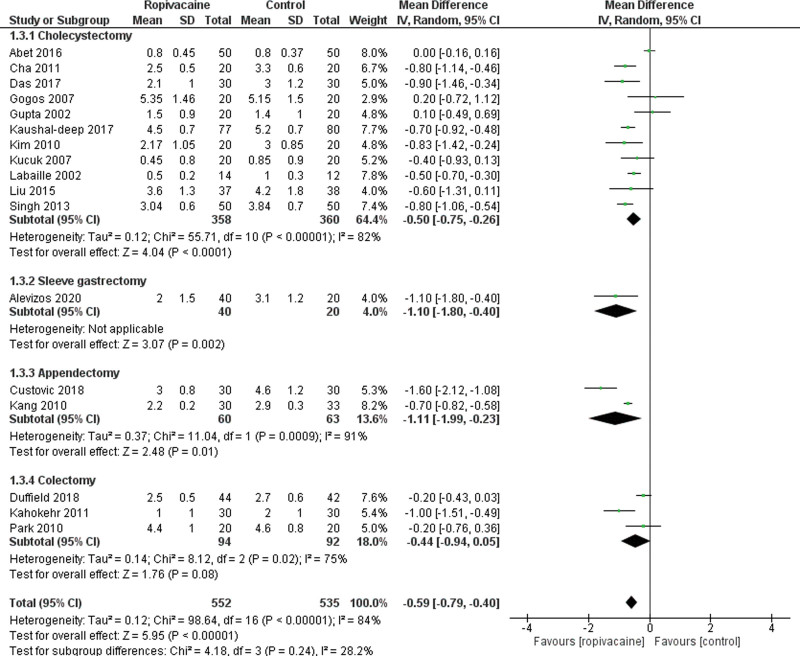
Forest plot for visual analogue scale at 8 hours. CI = confidence interval, SD = standard deviation.

Concerning the VAS score at H8, it was reported in 17 trials.^[14, 20–23, 25–27, 29, 31, 33, 35, 37, 39–41]^ They showed that VAS-H8 was lower in the intraperitoneal group with a significant difference (Moderate certainty of evidence; MD = −0.59 95% CI [−0.79, −0.40], *P* < .01). There was a low heterogeneity among the studies Tau^2^ = 0.12 (I^2^ = 84%; Fig. 4).

**Figure 5.**
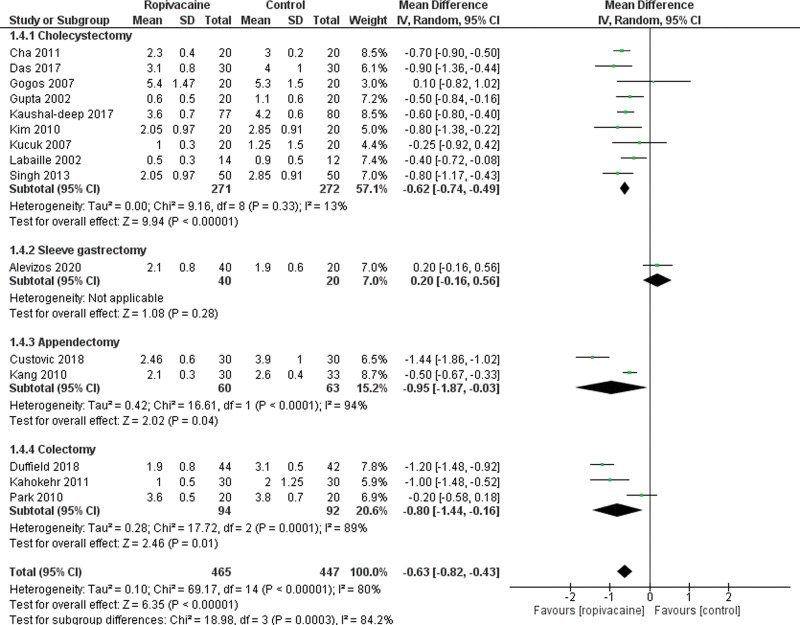
Forest plot for visual analogue scale at 12 hours. CI = confidence interval, SD = standard deviation.

**Figure 6.**
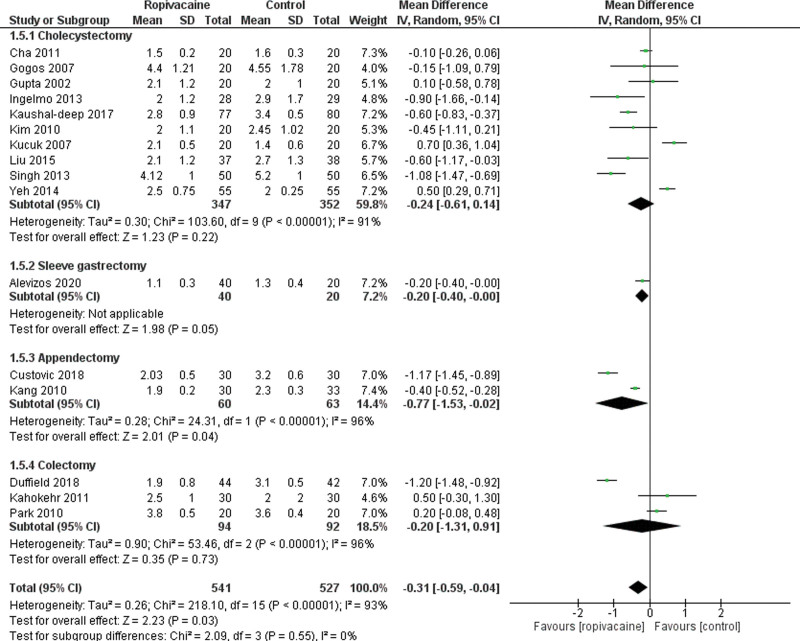
Forest plot for visual analogue scale at 24 hours. CI = confidence interval, SD = standard deviation.

Besides, 15 studies^[14, 21–23, 25, 26, 29, 31–33, 35, 37, 39–41]^ showed that the VAS score at H12 was lower in the intraperitoneal group compared to the control group (Moderate certainty of evidence; MD = −0.63 95% CI [−0.82, −0.43], *P* < .01), with a low heterogeneity among the trials Tau^2^ = 0.10 (I^2^ = 80%; Fig. 5). Similar results were seen concerning the VAS score at H24 with significantly lower data in the intraperitoneal group (MD = −0.31 95% CI [−0.59, −0.04], *P* < .01; Fig. 6).

#### 4.1.2. Postoperative nausea and vomiting

**Figure 7.**
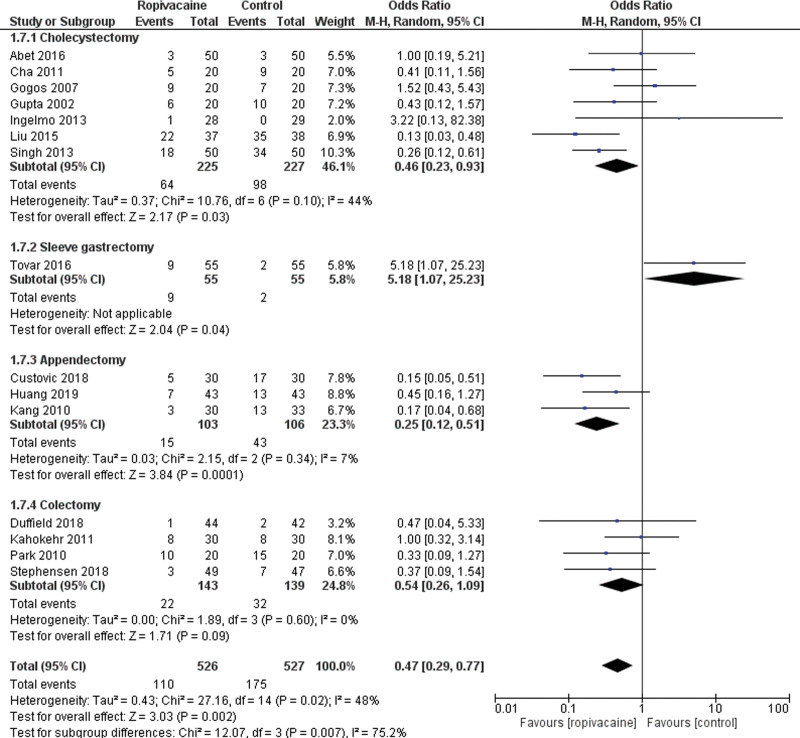
Forest plot for postoperative nausea and vomiting. CI = confidence interval.

A total of 15 studies assessed data on PONV.^[20, 21, 23, 24, 27, 29, 31, 34–37, 39–42]^ They included 526 patients in the intraperitoneal group versus 527 in the control group. Pooled results showed that intraperitoneal instillation of ropivacaine reduced the rate of PONV (Moderate certainty of evidence; MD = 0.47 95% CI [0.29, 0.77], *P* < .01). There was a low heterogeneity among the studies Tau^2^ = 0.43 (I^2^ = 48%; Fig. 7).

#### 4.1.3. Length of hospital stay

**Figure 8.**
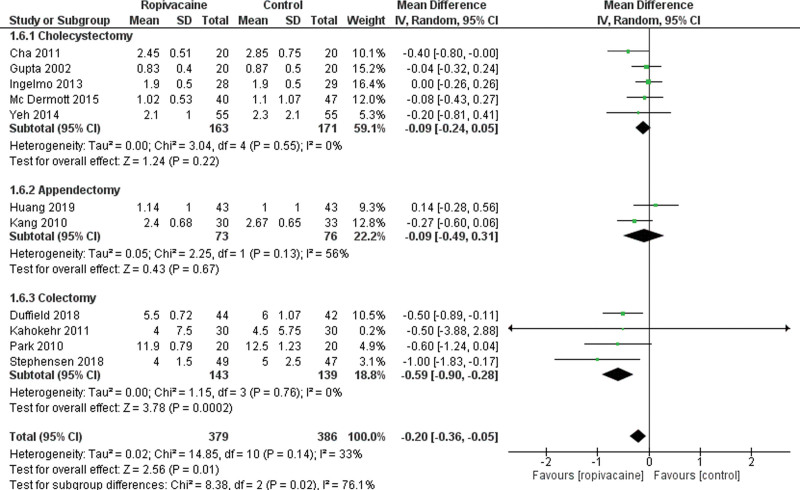
Forest plot for length of hospital stay. CI = confidence interval, SD = standard deviation.

Eleven studies reported data on LOS following LS.^[21, 23, 24, 28, 30, 36, 37, 39–42]^ We found a significantly shorter LOS in the intraperitoneal group (Moderate certainty of evidence; MD = −0.20 95% CI [−0.36, −0.05], *P* < .01) with a low heterogeneity among the included studies Tau^2^ = 0.02 (I^2^ = 33%; Fig. 8).

#### 4.1.4. Frequency of shoulder pain

**Figure 9.**
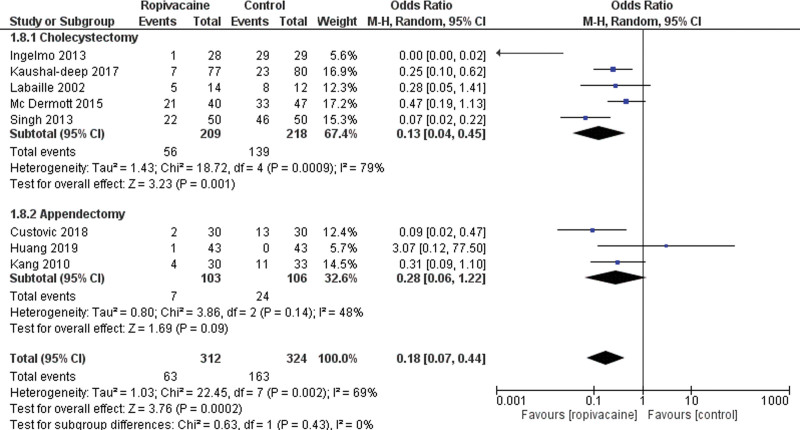
Forest plot of shoulder pain. CI = confidence interval.

This outcome was reported by 8 studies.^[14, 24, 26, 28, 31, 35–37]^ Sixty-three patients out of 312 reported shoulder pain in the intraperitoneal group versus 163 patients out of 324 in the control group (Low certainty of evidence; MD = 0.18 95% CI [0.07, 0.44], *P* < .01). There was high heterogeneity among the studies Tau^2^ = 1.03 (I^2^ = 69%). We performed a subgroup analysis comparing the cholecystectomy group and the appendectomy group. Five studies reported data on shoulder pain following cholecystectomy. There was a lower rate of shoulder pain in the intraperitoneal group (MD = 0.13 95% CI [0.04, 0.45], *P* < .01). Three studies reported data following an appendectomy and found the same results with a lower rate in the intraperitoneal group (MD = 0.28 95% CI [0.06, 1.22], *P* < .01; Fig. 9).

## 5. Summary of findings of the included studies and the effects of intraperitoneal ropivacaine instillation

A summary of the evidence is presented in Table 3. This review shows that the intraperitoneal ropivacaine instillation:

**Table 3:** Summary of findings table.

Outcomes	Number of participants (studies) Follow-up	Certainty of the evidence (GRADE)	Relative effect (95% CI)	Anticipated absolute effects	
				Risk with the control group	Risk difference with intraperitoneal ropivacaine
Opioid consumption	1078 (17 RCTs)	⨁⨁⨁◯ Moderate^*^	–	–	MD **21.93 lower** (27.64 lower to 16.23 lower)
VAS-H4	1547 (22 RCTs)	⨁⨁⨁◯ Moderate^*^	–	–	MD **1.05 lower** (1.31 lower to 0.79 lower)
VAS-H8	1087 (17 RCTs)	⨁⨁⨁◯ Moderate^*^	–	–	MD **0.59 lower** (0.79 lower to 0.4 lower)
VAS-H12	912 (15 RCTs)	⨁⨁⨁◯ Moderate^*^	–	–	MD **0.63 lower** (0.82 lower to 0.43 lower)
Hospital stay	765 (11 RCTs)	⨁⨁⨁◯ Moderate^†^	–	–	MD **0.2 lower** (0.36 lower to 0.05 lower)
PONV	1053 (15 RCTs)	⨁⨁⨁◯ Moderate^†^	**OR 0.47** (0.29–0.77)	332 per 1000	**143 fewer per 1000** (206 fewer to 55 fewer)
Shoulder pain	636 (8 RCTs)	⨁⨁◯◯ Low^*,†^	**OR 0.18** (0.07–0.44)	503 per 1000	**349 fewer per 1000** (437 fewer to 195 fewer)

GRADE Working Group grades of evidence – High certainty: We are very confident that the true effect lies close to that of the estimate of the effect. Moderate certainty: We are moderately confident in the effect estimate: the true effect is likely to be close to the estimate of the effect, but there is a possibility that it is substantially different. Low certainty: Our confidence in the effect estimate is limited: the true effect may be substantially different from the effect estimate. Very low certainty: We have very little confidence in the effect estimate: the true effect is likely to be substantially different from the estimate of effect.

CI = confidence interval, GRADE = Grading of Recommendations Assessment, Development and Evaluation, MD = mean difference; PONV = postoperative nausea and vomiting, OR = odds ratio, RCTs = randomized controlled trials, VAS = Visual Analogue Pain Scores.

The risk in the intervention group (and its 95% confidence interval) is based on the assumed risk in the comparison group and the relative effect of the intervention (and its 95% CI).

* *I*² > 50%.

† Small sample size.

- It probably leads to leads to a large reduction in opioid consumption.
- It probably reduces VAS-H4, VAS-H8, VAS-H12, hospital stay, and PONV.
- It may reduce postoperative shoulder pain.

## 6. Discussion

This systematic review and meta-analysis assess the efficacy and safety of ropivacaine intraperitoneal infusion (IR) following laparoscopic digestive surgery. Our findings indicate that IR is associated with reduced opioid consumption during the first 24 hours postoperatively and decreased VAS scores at various intervals. Additionally, it lessened PONV and the frequency of postoperative shoulder pain. Subgroup analysis demonstrated improved acute pain management across different procedures in the IR group.

Intraperitoneal infusion of ropivacaine has shown promising results in pain management post-surgery. Several studies have explored the efficacy and safety of intraperitoneal instillation of local anesthetics for postoperative pain management in various surgical procedures. Jain et al^[43]^ found that transversus abdominis plane block significantly decreased postoperative pain and opioid requirement in patients undergoing laparoscopic intraperitoneal onlay mesh repair. In a study by Kaur et al,^[44]^ the administration of ropivacaine intraperitoneally during laparoscopic bariatric surgery was shown to reduce postoperative pain in the recovery room. However, it did not significantly reduce opioid use or LOS. Jarrar et al,^[45]^ investigated the impact of intraperitoneal local anesthesia with ropivacaine on enhanced recovery after bariatric surgery outcomes and found that it did not reduce postoperative pain or analgesic consumption in patients undergoing laparoscopic Roux-en-Y gastric bypass surgery. Furthermore, Randa et al^[46]^ conducted a randomized clinical trial comparing intraperitoneal local instillation of levobupivacaine, magnesium sulfate, and a combination of both for postoperative pain relief after laparoscopic sleeve gastrectomy. The study aimed to assess the efficacy of different analgesic agents in managing postoperative pain in this specific surgical procedure. Overall, the literature suggests that intraperitoneal instillation of local anesthetics, including ropivacaine, can be effective in reducing postoperative pain in various laparoscopic surgeries. However, the impact on opioid use and LOS may vary depending on the specific surgical procedure and patient population. Previous systematic reviews by MacFaster et al^[47]^ and Hamil et al^[48]^ highlighted IR’s positive analgesic effects after open and laparoscopic surgeries. However, significant heterogeneity was noted due to the inclusion of various abdominal surgeries and local anesthetics with differing pharmacokinetics. Limiting the inclusion of RCTs that used ropivacaine as the sole local anesthetic in laparoscopic digestive surgeries was intended to minimize heterogeneity and enhance evidence quality. The peritoneum, a bilayer of flat epithelial cells,^[49]^ richly innervated by the phrenic nerve, spinal segment nerves, and the vagus nerve, provides an anatomical basis for the direct analgesic effects of local anesthetics. Since 1950,^[50]^ the intraperitoneal infusion of local anesthetics has been explored to diminish opioid usage and shoulder pain frequency post-laparoscopy, yet their effectiveness remains debated. Our study confirms that ropivacaine’s intraperitoneal infusion significantly reduces opioid consumption, PONV, and shoulder pain incidence postoperatively. Following concerns over bupivacaine toxicity, ropivacaine, a long-acting local anesthetic with lower cardiotoxicity and reduced central nervous system toxicity, was developed.^[51]^ Despite ropivacaine’s pharmacokinetics suggesting a duration of action not exceeding 1 day, our research observed a notable reduction in pain scores 24 hours after surgery. The effect size was reduced by half from 4 hours (1.05) to 8 hours (0.59), aligning with findings from Gurusamy et al,^[52]^ who reviewed 48 RCTs on intraperitoneal local anesthetic instillation in laparoscopic cholecystectomy. Clinically significant pain score changes require a minimum shift of 1 to 1.7 on a 10-point scale,^[53–55]^ emphasizing the importance of parameters like opioid consumption and shoulder pain frequency over mere pain scores. Limited data on wound infection and intra-abdominal collections suggest ropivacaine’s intraperitoneal instillation as a safe regional analgesic technique, supported by Yong et al,^[6]^ who reported fewer adverse events in the intraperitoneal group compared to controls.

In considering the broader implications of our findings, it is essential to explore the potential impact of intraperitoneal ropivacaine instillation on the global opioid crisis. The current study highlights a significant reduction in opioid consumption among patients receiving ropivacaine, pointing towards a viable strategy for minimizing opioid reliance post-surgery. This approach not only aligns with the goals of enhanced recovery after surgery protocols to optimize postoperative outcomes but also offers a critical tool in addressing the escalating concerns related to opioid overuse, misuse, and dependency, which have been declared a public health emergency in several countries. By providing an effective non-opioid analgesic alternative, intraperitoneal ropivacaine instillation could contribute to the paradigm shift in postoperative pain management strategies, potentially reducing the incidence of chronic pain and the long-term socio-economic burdens associated with opioid addiction.

This review and meta-analysis acknowledge certain limitations, including the exclusive inclusion of RCTs, resulting in a small sample size and inconclusive evidence. Despite attempts to address high heterogeneity through subgroup analysis, it persisted across some outcomes. The heterogeneity could not be fully explained by the instillation technique or timing, nor by the variety of ropivacaine applications. These factors necessitated the use of the 5-point Cochrane Handbook recommendation and the CONSORT statement to assess and mitigate the risk of bias and improve study quality assessment. Although efforts were made to standardize outcome reporting, some outcomes were poorly defined or unmeasured, particularly regarding adverse effects. Therefore, these findings should be interpreted with caution and validated through multicenter RCTs.

## 7. Conclusion

Our study demonstrates that intraperitoneal instillation of ropivacaine significantly reduces total opioid consumption after digestive surgery, enhances postoperative recovery by decreasing PONV, shortens hospital stays, and lowers shoulder pain frequency, making it a valuable component of the Enhanced Recovery After Surgery protocol. However, the absence of comprehensive safety data in the literature precludes a definitive assessment of this technique’s safety profile. Further high-quality trials are essential to confirm the efficacy and safety of ropivacaine’s intraperitoneal instillation as part of a multimodal pain management approach to enhance patient comfort.

## Author contributions

**Conceptualization:** Mohamed Aziz Daghmouri, Mohamed Ali Chaouch, Benjamin Deniau, Laurent Benayoun, Bassem Krimi, Amine Gouader.

**Data curation:** Mohamed Aziz Daghmouri, Mohamed Ali Chaouch, Amine Gouader, Hani Oweira.

**Formal analysis:** Mohamed Ali Chaouch, Bassem Krimi.

**Funding acquisition:** Mohamed Aziz Daghmouri.

**Investigation:** Mohamed Aziz Daghmouri, Amine Gouader.

**Methodology:** Mohamed Aziz Daghmouri, Benjamin Deniau, Hani Oweira.

**Project administration:** Amine Gouader.

**Resources:** Benjamin Deniau, Bassem Krimi.

**Software:** Mohamed Aziz Daghmouri, Mohamed Ali Chaouch, bassem Krimi, Amine Gouader, Hani Oweira.

**Supervision:** Laurent Benayoun, Hani Oweira.

**Validation:** Mohamed Ali Chaouch, Benjamin Deniau, Laurent Benayoun, Bassem Krimi, Amine Gouader, Hani Oweira.

**Visualization:** Hani Oweira.

**Writing – original draft:** Mohamed Aziz Daghmouri, Mohamed Ali Chaouch, Benjamin Deniau, Bassem Krimi, Amine Gouader.

**Writing – review & editing:** Laurent Benayoun, Hani Oweira.

## Supplementary Material


