# Supplementary material for: Efficacy and safety of intraperitoneal ropivacaine in pain management following laparoscopic digestive surgery: A systematic review and meta-analysis of RCTs
Source: Medicine (Baltimore). 2024 Jul 19;103(29):e38856. doi: 10.1097/MD.0000000000038856 (PMC11398749; doi:10.1097/MD.0000000000038856)
Supplement: Supplementary file 1 [file medi-103-e38856-s001.pdf]

Risk of bias domains

	D1	D2	D3	D4	D5	Overall
Abet et al	X	-	+	+	+	X
Alevizos et al	X	-	+	+	-	X
Cha et al	+	+	+	+	+	+
Custovic et al	-	X	+	+	+	-
Das et al	+	+	+	+	+	+
Duffield et al	+	+	+	+	+	+
Gogos et al	X	X	+	+	+	X
Gupta et al	X	X	+	+	+	X
Gogos et al	X	X	+	+	+	X
Gupta et al	X	X	+	+	+	X
Gogos et al	X	X	+	+	+	X
Gupta et al	X	X	+	+	+	X
Gogos et al	X	X	+	+	+	X
Gupta et al	X	X	+	+	+	X
Gogos et al	X	X	+	+	+	X
Gupta et al	X	X	+	+	+	X
Gogos et al	X	X	+	+	+	X
Gupta et al	X	X	+	+	+	X
Gogos et al	X	X	+	+	+	X
Gupta et al	X	X	+	+	+	X
Gogos et al	X	X	+	+	+	X
Gupta et al	X	X	+	+	+	X
Gogos et al	X	X	+	+	+	X
Gupta et al	X	X	+	+	+	X
Gogos et al	X	X	+	+	+	X
Gupta et al	X	X	+	+	+	X
Gogos et al	X	X	+	+	+	X
Gupta et al	X	X	+	+	+	X
Gogos et al	X	X	+	+	+	X
Gupta et al	X	X	+	+	+	X
Gogos et al	X	X	+	+	+	X
